# Adapting Health Services in Forced Displacement: Operationalizing Surge Capacity Framework in the EMT Barco San Raffaele, Colombia

**DOI:** 10.3390/ijerph23040435

**Published:** 2026-03-30

**Authors:** Lina Echeverri, Ana Lucia Lopez, Diego Orlando Posso, Ives Hubloue, Luca Ragazzoni, Flavio Salio

**Affiliations:** 1 CRIMEDIM (Centre for Research and Training in Disaster Medicine, Humanitarian Aid and Global Health), Università del Piemonte Orientale, 28100 Novara, Italy; 2 EMT Barco San Raffaele, Cali 760001, Colombiapresidencia@barcohospitalhsr.org (D.O.P.); 3 Research Group on Emergency and Disaster Medicine, Medical School, Vrije Universiteit Brussel, 1050 Brussel, Belgium; 4 Global Health Emergency Corps, World Health Organization (WHO), 1211 Geneva, Switzerland; saliof@who.int

**Keywords:** mixed migration, forced displacement, conflict, urban violence, migration crises, adaptation of health services, cross border displacement

## Abstract

**Highlights:**

**Public health relevance—How does this work relate to a public health issue?**

- Forced displacement represents both an acute emergency and a structural global health challenge, driving persistent health inequities, disrupting continuity of care, and increasing vulnerability among migrant, refugees and internally displaced populations.
- This study examines how health services in emergency contexts can be systematically adapted to local realities to address the needs of the forcibly displaced population (internal or refugee influx) while operationalizing global commitments to migrant health, equity, and universal health coverage.

**Public health significance—Why is this work of significance to public health?**

- This research is novel in applying the WHO EMT Surge Capacity Framework (Staff, Structure, Supplies, Systems) to adapt and guide health service adaptation in displacement crises.
- Drawing on Global South operational experience, the findings demonstrate how flexible platforms, culturally informed care, workforce training, and digital innovations can simultaneously address emergency needs and strengthen long-term system resilience.

**Public health implications—What are the key implications or messages for practitioners, policy makers and/or researchers in public health?**

- Emergency responses to displacement must move beyond short-term humanitarian relief toward integrated, system-strengthening approaches that ensure equitable access and continuity of care across mobility trajectories.
- Operational flexibility, including mobile infrastructure, telemedicine, and navigating relevant legislations such as IHL, IHRL, Refugee Law, and domestic legislation, is essential to uphold the right to health and respond effectively to displacement as a sustained global health challenge.

**Abstract:**

(1) Background: Colombia hosts one of the world’s largest mixed-displacement crises, combining longstanding internal displacement with the influx of Venezuelan migrants. This case study examines how the Emergency Medical Team (EMT) Hospital Barco San Raffaele (HBSR) adapted its service-delivery model to respond simultaneously to internal displacement in the Colombian Pacific region and the Venezuelan refugee influx. Using the WHO EMT Surge Capacity Framework, the study analyses how health services were adapted across two concurrent displacement contexts. (2) Methods: A mixed-methods comparative case study was conducted using mission reports, epidemiological surveillance data, policy reports and institutional documents collected between November 2020 and May 2021. Data were analyzed through a thematic analysis structured around the four domains of the WHO EMT Surge Capacity Framework (Staff, Structure, Supplies and Systems), to examine how service adaptation was operationalized across different geographic, sociocultural and legal environments; (3) Results: EMT HBSR adapted staffing composition, supply chains, infrastructure, and operational systems across both settings. Its hybrid model, combining a hospital boat platform with mobile outreach teams, enabled continuity of primary care, mental, maternal and child health, and community-based services in geographically isolated and culturally diverse communities; (4) Conclusions: The findings illustrate how flexible EMT operational models can support the adaptation of health services, and reduce health access inequalities in displacement contexts characterized by high mobility, confinement and limited health system capacity. Mobile platforms, such as hospital boats, appear to be a viable strategy for ensuring continuity of care along migratory routes and in geographically isolated areas affected by protracted instability.

## 1. Introduction

Migration and forced displacement [1] are reshaping cities, regions and countries [2]. These movements occur both across international borders and within States, and may result from armed conflict, generalized violence, serious violations of human rights, human-made or natural disasters [3], events seriously altering public order [2].

The number of people displaced by conflict and natural disasters continues to rise, with climate change increasingly contributing to both migration and conflict dynamics [2]. This cycle of displacement is intensified by poverty, weak governance, unplanned urbanization, conflict and climate-related risks. These interconnected challenges weaken community resilience [4] and strain health systems, limiting their capacity to prepare for and respond to health crises.

Forced displacement settings sustained pressure on health systems operating in fragile and resource-constrained environments. In protracted and acute crises, the ability of health institutions to maintain essential services depends on their capacity to foresee and absorb shocks, reorganize resources, and adapt service delivery models. This adaptive capacity is described as health system resilience [5].

According to UNHCR, the number of forcibly displaced people has doubled over the last decade [6], rising from 59.5 million by the end of 2014 to 123.2 million by the end of 2024 [7]. Over the last five years, internal displacement has grown by 51%, reaching 75.9 million by the end of 2023, many of whom have experienced displacement for prolonged periods [8]. Without lasting solutions, displacement figures are likely to keep rising, reinforcing forced displacement as one of the most urgent humanitarian challenges and a powerful driver of contemporary migration [4].

Human mobility is often framed as a security concern [9]. The relationship between migration and security is complex and multidimensional. Both terrorism and counter-terrorism efforts can contribute to displacement internally or across borders. Heightened border controls, detention, and unlawful returns of refugees or “pushbacks” often result in increased human rights violations [10].

Furthermore, terrorism’s cross-border nature significantly influences migration policy [11]. Weak policy coordination and conflicting policy objectives [9] can exacerbate risks, heighten vulnerabilities and worsen the health challenges faced by forcibly displaced populations. Health policies operate within this same landscape.

In some contexts, migration is framed as a public health threat, fueling restrictive measures; in others, a rights-based approach prevails, recognizing the multiple vulnerabilities of migrants and displaced persons and emphasizing equitable access to essential health services as a core component of universal health coverage [9]. These interrelated challenges have prompted the development of global policy responses aimed at safeguarding the health rights of migrants and displaced populations, while promoting equitable access to care.

In fragile and complex humanitarian settings, the effectiveness of health responses depends not only on availability of resources and adaptative capacity, but also on governance arrangements that shape and improve coordination and collective actions between humanitarian actors, national authorities, development and peace institutions. This integrated approach addressing complex challenges in conflict-affected and protracted settings is known as the humanitarian-development-peace nexus, aiming to ensure collective and coherent outcomes between emergency response and long-term system strengthening [12].

The World Health Assembly resolution WHA61.17 [13] identifies migrant health as a key public health concern and calls for regional and national health strategies ensuring equitable access to services. Similarly, the WHO Emergency Medical Teams (EMT) Initiative, in its 2030 Strategy, aligns with the WHO Global Health for Peace Initiative (GHPI), prioritizes service delivery in conflict-affected and vulnerable settings, and commits to developing minimum technical standards for migration crises [14,15,16,17].

Despite growing attention to migration health and displacement, important gaps remain in understanding how health services are operationally adapted in acute and protracted displacement contexts, and the influence of legal frameworks on access to care. While frameworks such as the WHO Emergency Medical Teams (EMT) Surge Capacity provide guidance for emergency response, empirical evidence remains limited on how these mechanisms are operationalized in settings characterized by prolonged displacement, mixed migration flows, and constrained health system capacity.

In this context, this research focuses specifically on forced displacement through an analytical case study, where Colombia provides a relevant setting for examining these dynamics. To situate the analysis, key migration-related definitions are summarized in Table A1.

Colombia has experienced a prolonged low-intensity asymmetric conflict since the 1960s, involving state forces, far-left guerrillas, far-right paramilitary groups, and drug cartels. Despite recent reductions in violence [18], eight active Non-International Armed Conflicts (NIACs) persist, generating a sustained humanitarian crisis marked by displacement, confinement and movement restrictions across multiple regions [19]. Indigenous and Afro-descendant communities account for over half of confined populations, highlighting disproportionate vulnerability, loss of autonomy, and social fragmentation [20].

At the same time, Colombia is at the center of the world’s second-largest displacement crisis after Ukraine. The country hosts nearly 3 million Venezuelan migrants, representing around 40% of the regional total, while also confronting 7.3 million internally displaced Colombians [21,22]. These flows include Colombian returnees and Venezuelan migrants with diverse legal statuses. Many face high levels of vulnerability and limited documentation, reflecting patterns observed in other global forced-displacement crises and creating layered humanitarian and development challenges [23].

These pressures are further compounded by the escalating Darién Gap crisis, a major jungle corridor between Colombia and Panama that was crossed by more than 500,000 people in 2023 alone [24]. Migrants from multiple countries face severe environmental hazards, violence, and health risks along this route, placing significant governance, security, and humanitarian pressures on Colombia and the wider region [20]. The convergence of refugees, returnees, pendular migrants, transit migrants, and IDPs illustrates the scale and complexity of Colombia’s mixed-migration landscape. Despite these challenges, Colombia has implemented progressive measures to regularize status and expand access to health, education, housing, and livelihoods [23].

### 1.1. The EMT Initiative in Colombia and the Uniqueness of the EMT Hospital Barco San Raffaele

Colombia’s Ministry of Health and Social Protection issued the Resolution No. 00000633 on 15 April 2024 [25], which officially adopts the WHO Emergency Medical Teams (EMT) Initiative as a national program for risk reduction and health emergency responses. The resolution builds on Colombia’s recurrent exposure to natural and human-induced disasters, which have repeatedly overwhelmed health services across the country. In alignment with the Sendai Framework for Disaster Risk Reduction 2015–2030 and the Pan American Health Organization (PAHO) regional implementation program, Colombia seeks to strengthen its national health emergency response capacities through the establishment of a formal system for EMTs registration, certification, coordination, and continuous monitoring and evaluation, ensuring standardized, harmonized and high-quality medical response to emergencies, particularly those affecting remote or hard-to-reach communities.

The adoption of the EMT Initiative represents a significant milestone for Colombia, as it institutionalizes an internationally recognized framework that enhances the professionalization, coordination, and accountability of medical response teams. In this context, the EMT BHSR, a non-profit organization launched in 2009, is designed to bring essential healthcare services to population without access to medical infrastructure across the country with particular focus on the Pacific Region [26].

This floating hospital travels coastal areas and rivers for about 10–12 days each month, overcoming structural and geographic barriers to healthcare access, especially for communities affected by armed conflict. Unlike vessels designed for short-term search and rescue missions, the EMT BHSR operates as a floating, flexible healthcare platform that delivers continuous and specialized outpatient services. It provides general medicine with a family-centered approach, pediatrics, internal medicine, gynecology/obstetrics, surgery, anesthesia, and dentistry, supported by laboratory and imaging services. Each mission offers medical care to approximately 3000–3500 people per month, reaching around 24,000 beneficiaries annually.

Its model of care, rooted in Health Risk Management and Primary Health Care (PHC) [27], integrates prevention, timely treatment, and community engagement through an *intercultural approach* as a central pillar that respects Afro-Colombian and Indigenous knowledge. The ship also strengthens public health surveillance and community resilience by collecting real-time epidemiological data to inform national health strategies, a capacity that proved invaluable during the COVID-19 pandemic. Furthermore, it has trained over 700 traditional midwives across four departments (Chocó, Valle del Cauca, Cauca, and Nariño), expanded access to family planning for around 200 women per month, and provided psychosocial and medical support to approximately 100–150 victims of gender-based violence each month.

Through this integrated, community-based, and culturally sensitive model, the Barco Hospital has become a cornerstone of health service delivery and system resilience in Colombia’s Pacific region, embodying a long-standing commitment to equitable access and strengthening local capacity well before its formal classification as an Emergency Medical Team (EMT) Type 1 in September 2024.

### 1.2. Study Rationale and Objective

Although the WHO Emergency Medical Teams (EMT) Surge Capacity model has demonstrated value in emergency preparedness and response, its application in forced displacement crises remains insufficiently examined as a framework for health service adaptation. This gap constrains the ability of national health systems, local authorities, and medical teams to anticipate and respond to the complex health needs of forcibly displaced populations, which often require coordinated and flexible service delivery models.

This study examines the experience of the EMT Barco Hospital San Raffaele (BHSR) in responding to two displacement crises in Colombia. Using the WHO EMT Surge Capacity domains as an analytical framework, the study explores how health services were adapted across different operational contexts and identifies practices that may strengthen the adaptability of medical teams responding to forced displacement settings.

## 2. Methods, Intervention and Comparative Analysis

The EMT BHSR represents a distinctive EMT service model that combines a floating health platform with onboard clinical infrastructure and mobile outreach teams, enabling flexible service delivery in remote and crisis-affected settings. Its model also integrates intercultural engagement with Afro-Colombian and Indigenous communities, real-time epidemiological data collection, and context-specific adaptation of care. This study offered a unique opportunity for comparative analysis because EMT BHSR responded simultaneously to two forced displacement crises in the same country but involving different populations and operational conditions. The Venezuelan crisis was associated mainly with political, social, and economic instability and supported through humanitarian corridors, while internal displacement in Colombia reflected a protracted crisis characterized by armed conflict, confinement, and restricted mobility. Analyzing these two responses in parallel allowed a better understanding of how health services were adapted under contrasting displacement dynamics.

### 2.1. Study Design and Analytical Framework

This study used a mixed-methods, cross-sectional, comparative case study design to examine how the EMT BHSR adapted health services while responding concurrently to two forced displacement events in Colombia: internal displacement in the Pacific Region and the Venezuelan forced displacement crisis. This design allowed comparison across two distinct humanitarian settings within the same operational period.

The WHO EMT Surge Capacity Framework [28,29] served as the main analytical lens and informed the predefined themes used in the qualitative analysis. Its four domains: *Staff*, *Supplies*, *Structure and Systems*, were used as a priori themes to examine how service adaptations were implemented across varying geographic, legal, and sociocultural constraints. In addition, a continuum-of-care perspective was applied to identify where adaptations occurred along the service delivery pathway.

The decision to apply the surge capacity domains was guided by two considerations. First, the EMT Initiative positions the surge capacity as a core pillar of health system readiness and prioritizes it for capacity strengthening [29]. Second, despite its central role within the EMT doctrine, the Surge Capacity Framework has not been systematically operationalized in health responses in forced displacement settings, nor has it been widely used to analyze service adaptation in such contexts.

### 2.2. Data Sources and Selection Criteria

Data sources included EMT BHSR mission reports, epidemiological surveillance data, institutional documents, policy reports, and relevant literature. Primary data comprised EMT BHSR operational mission reports and epidemiological surveillance records generated during service delivery. Secondary data included institutional documents, policy materials and literature relevant to migration, forced displacement and access to health services in Colombia.

Materials were included if they related to either of the two displacement events, fell within the study period (November 2020 to May 2021), and contained information on service delivery, epidemiological trends, operational constraints, coordination, or surge capacity. The period was selected because EMT BHSR supported the government response to the Venezuelan forced displacement crisis while concurrently addressing internal displacement in the Colombian Pacific Region. Materials not meeting these criteria were excluded.

### 2.3. Data Extraction and Comparative Analysis

Data were organized using a structured extraction matrix. Quantitative data were assessed for completeness and consistency, then analyzed descriptively to compare service delivery and epidemiological trends across the two contexts. Qualitative data were drawn from mission reports, policy documents and context-relevant literature, and analyzed using the four EMT Surge Capacity domains as predefined themes.

Data were subsequently synthesized into a combined analytical matrix linking: (1) operational challenges or health needs; (2) implemented service adaptations; (3) surge capacity domains; and (4) operational implications across the continuum of care. This approach enabled a structured comparison of how EMT BHSR adapted service delivery across the two displacement events. Triangulation was conducted by comparing findings across mission reports, epidemiological surveillance data, institutional documents and policy reports. This facilitated cross-validation of patterns identified in individual sources and supported consistency in interpretation across both case contexts.

**Figure 1:**
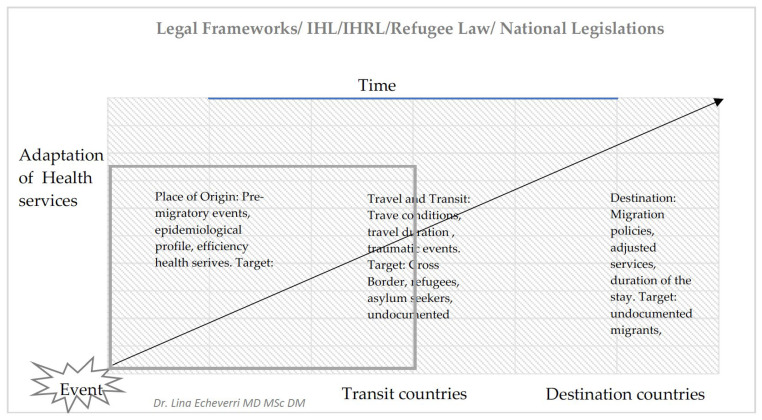
From origin to destination: health system readiness and response in forced displacement contexts.

Figure 1 presents the conceptual framework for the study, situating health service adaptation along the migration cycle and across varying levels of response capacity. Within this framework, the analysis examined how the surge capacity domains were operationalized and adapted to maintain access to care under geographic, legal, and sociocultural constraints. Legal and regulatory conditions were treated as contextual factors shaping service adaptation, including international and domestic legal frameworks that could enable or constrain access to care and influence the scope of medical teams’ interventions over time.

### 2.4. Legal, Bias and Ethical Considerations

Legal and regulatory frameworks, including International Humanitarian Law, International Human Rights Law, Refugee Law, and domestic regulations, were treated as cross-cutting contextual factors influencing access to care and the scope of service adaptation.

As the study relied mainly on organizational data from EMT BHSR, and some authors had institutional links to the organization, potential interpretative bias was mitigated using multiple data sources, structured data extraction, and triangulation. Formal independent coding was not undertaken; however, analytical consistency was supported by predefined themes based on the EMT Surge Capacity domains and triangulation across the data sources described above.

The study involved retrospective analysis of routinely collected, non-identifiable operational, epidemiological data, as well as policy and literature sources. No direct interaction with patients or affected populations was conducted for the purpose of this research.

## 3. Results

### 3.1. Venezuelan Forced Displacement Intervention

Colombia’s response to the Venezuelan crisis evolved through three phases: an initial humanitarian phase (2015–2017), focused on receiving Colombian returnees and the first wave of Venezuelan migrants; a second phase (2018–2021), centered on coordinated medium-term measures and expanding migrants access to basic services such as health, education, and social protection; and the third ongoing phase (since 2021), aimed at long-term integration through mass regularization, access to markets and services, and social cohesion policies [23].

**Graph 1:**
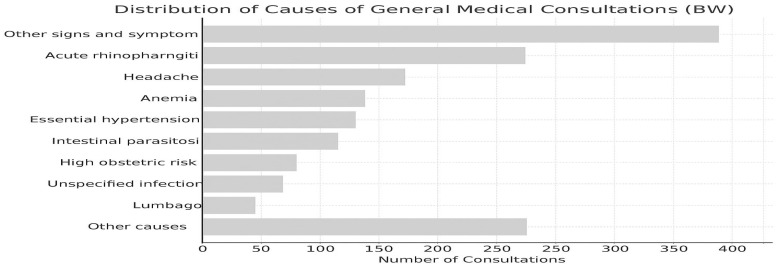
Distribution of causes (source: EMT Barco Hospital San Raffaele).

During the response period, EMT BHSR delivered integrated clinical, mental health, and public health services in southwest Colombia, focusing on a designated sector of the humanitarian corridor under the overall coordination of the Colombian health authorities. Its intervention targeted migrants in transit within this operational area and did not cover the northeastern cross-border influx. As shown in Graph 1, the clinical profile was highly variable requiring flexible, day-to-day service planning. High demand was observed for dental care, likely associated with poor hygiene access during migration journeys, and for long-term contraceptive methods, particularly subdermal implants. Disease-specific treatment kits and laboratory testing were introduced to support detection and management of notifiable infections, including HIV and syphilis. These findings illustrate the importance of adaptable service packages capable of addressing, simultaneously, acute, chronic, preventive, and sexual and reproductive health needs among mobile populations.

Initial resistance to mental health services was driven by fear of deportation, reprisals, mistrust regarding misuse of personal data and prior experiences of violence. Through community engagement and collaboration with migrant community leaders and educational campaigns, trust gradually increased. This made it possible to identify previously unmet needs, including trauma, depression, and sexual abuse, including among children. Interagency collaboration enabled distribution of dignity kits and food, reducing tensions and improving acceptance of psychosocial services. This experience highlights the operational importance of trust-building, cultural mediation, and community engagement in the delivery of mental health and protection-related services.

**Graph 2:**
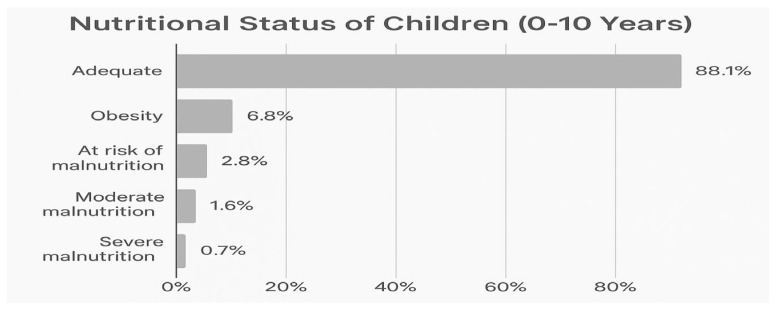
Nutritional assessment in children (source: EMT BHSR).

Pediatric consultations showed that 88% of children met WHO growth standards, while 11.9% presented malnutrition or obesity (Graph 2). Common morbidities included acute rhinopharyngitis (137 cases), intestinal parasitosis (89 cases), and dermatological mycoses (69 cases). Low vaccination coverage was noted among infants born in 2021. Nutritional counseling, deworming, and micronutrient supplementation were provided. A total of 99 pregnant women were attended, including 2 under 14 years and 17 between 15 and 17 years, indicating significant adolescent pregnancy vulnerabilities. Most pregnant women were aged 18–29 years (61 cases). Young and adolescent mothers were referred for prenatal monitoring and psychosocial support, underscoring the importance of integrated SRH and protection pathways. Concurrently, individual and group sessions on sexual and gender-based violence (SGBV) reached 1773 people (1468 women and 305 men), covering types of violence, prevention strategies and referral pathways, thereby reinforcing awareness and early reporting mechanisms. Printed information materials were also distributed.

Key operational challenges included the restrictions due to the COVID-19 pandemic, high population mobility, hindering accurate planning and monitoring, team security, community-level social barriers which varied from obstruction from some local leaders to misinformation and stigma, and weak inter-agency collaboration. To address these constraints, the EMT adapted schedules, strengthened daily debriefings, and promoted health education and community participation. These strategies gradually improved trust, visibility, and service uptake.

### 3.2. Internal Forced Displacement Intervention

Between November 2020 and May 2021, the EMT BHSR conducted multiple medical missions to the Pacific region, serving Afro-Colombian and Indigenous communities affected by armed conflict, confinement, and thus internally displaced. These missions were delivered in parallel with the Venezuelan response. In this context, EMT BHSR became a critical access point for populations facing prolonged isolation and mobility restrictions, with near-complete disruption of primary care services during the COVID-19 pandemic.

Communicable diseases were prominent due to overcrowding, poor hygiene, and limited access to safe water. Frequent conditions included scabies (21%), bacterial diarrhea, intestinal parasitosis, acute rhinopharyngitis, and tuberculosis. In response, teams integrated hygiene promotion, community health education, and distribution of hygiene and safe-water storage kits. Although the intervention took place during the COVID-19 pandemic, reported COVID-19 cases were low, likely reflecting community isolation and confinement. These findings show how the epidemiological profile required simultaneous adaptation of both clinical and public health functions.

Uncontrolled and decompensated chronic conditions were frequent, including anemia due to food insecurity, hypertension and diabetes due to prolonged interruption of medications. Medical teams reinforced supplies and adjusted treatment plans to account for interrupted care.

Sexual and reproductive (SRH) vulnerabilities were severe. Barriers to contraception increased unintended and adolescent pregnancies. Forty-nine percent (49%) of pregnant women visited were younger than 20, including cases under 14. The risk of sexual and gender-based violence (SV/GBV) was high. The EMT BHSR provided individual and group sessions to 1773 people on types of violence, reporting pathways, prevention strategies, and access to emergency SRH and STI treatment. High-risk pregnancies were the second leading cause of consultation, with women lacking antenatal care, laboratory tests, micronutrient supplementation, vaccines and the ability to move safely outside their territories. These findings point to the need for integrated maternal health, protection, and referral pathways in conflict-affected displacement settings.

Among 3456 pediatric consultations (0–10 years), 11.9% children presented nutritional conditions ranging from moderate or severe malnutrition to obesity; dental diseases were widespread due to lack of hygiene supplies and limited parental guidance. Vaccination gaps were significant, particularly among infants born in 2021.

Food insecurity was severe across all communities. Blockades by armed groups, mobility restrictions, including pandemic-related, and economic paralysis disrupted access to food. In response, teams distributed approximately 4000 food kits and provided micronutrient supplements (vitamin A, iron, and zinc) to children and pregnant women. Nutritional surveillance was implemented to detect moderate to severe acute malnutrition.

Mental health needs were significant, with widespread anxiety, depression, and post-traumatic stress linked to violence, confinement, and displacement. Community mistrust, driven by fear of reprisals and concerns over information misuse, limited early uptake of services. Through engagement with community leaders and culturally adapted communication strategies, teams gradually rebuild trust and improved participation in group-based psychological activities supporting emotional recovery.

The Barco Hospital faced unique operational challenges: (I) reconfiguration of the onboard infrastructure of the boat to allow physical distancing and reducing the workforce to comply with COVID-19 IPC measures, as communities restricted disembarkation and required all care to occur on the boat; (II) armed-groups-imposed working hours, patient flows and referral options, requiring continuous negotiation to maintain service access and continuation of care; (III) limited referral options for priority cases, due to structural limitation of the local health system; (IV) need to expand logistics and supplies capacity to overcome the interruption of local health services, particularly medicines for chronic diseases, food and hygiene kits, micronutrients, and treatment for SRH-SGBV related conditions; and (V) overcoming fear and mistrust related to mental health and SRH, necessitating sustained community engagement.

### 3.3. Comparative Analysis Using the Surge Capacity Domains 4 (S)

This section examines how EMT BHSR adapted health services across the two forced displacement crises using the four domains of the surge capacity (Table 1). A comparative analysis was conducted by triangulating mission reports, epidemiological and surveillance data, policy reports and institutional data from both crisis settings. Findings were interpreted against the WHO EMT Type 1 standards of care to identify alignments, gaps and context-specific variations.

Table 1 presents the comparative application of surge capacity domains in the Venezuelan forced displacement crisis and the internal displacement in Colombia’s Pacific region. The findings indicate that adaptation extended beyond isolated clinical adjustments, encompassing coordinated changes in services, staffing, infrastructure, supplies, and coordination systems. In the Venezuelan context, adaptation was strongly shaped by high population mobility, variable uptake of mental health services, and the need for flexible public health and sexual and reproductive health interventions for migrants in transit or with irregular status. By contrast, the Pacific response required deeper structural adaptation to geographic isolation, confinement, weak referral pathways and conflict-related protection constraints. Across both settings, continuity of care depended on the integrated operationalization of all surge capacity domains.

Table 2 compares the volume and type of health services delivered between November 2020 and May 2021. The Pacific/internal displacement response required a substantially higher number of general consultations, laboratory tests, maternal care, and community-based interventions, reflecting severe access barriers, greater health system disruption, and higher vulnerability linked to conflict and isolation. In contrast, the Venezuelan response showed proportionally greater emphasis on public health education, chronic disease management, psychosocial support and dental care. These differences indicate that service demand and staff configuration were strongly shaped by the epidemiological, social and operational characteristics of each displacement context.

These differences reflect the distinct operational and population dynamics of the two responses. In the Venezuelan crisis, EMT BHSR operated within a sectorized, inter-agency response in southwest Colombia, and the reported activities represent only the migrants directly attended by the team in its assigned areas, not the entire Venezuelan population. Most migrants were in transit along humanitarian corridors and typically seen only once during short stops, limiting the scope for continuity-based interventions. By contrast, the Pacific response targeted internally displaced communities living under confinement, geographic isolation, and prolonged disruption of local health services, requiring a broader and more sustained package of clinical, public health with community-based interventions. Notably, the absence of recorded psychosocial support activities in the Pacific region during the study period likely reflects contextual and documentation constraints, such as protection barriers, fear of reprisals, and concerns about the misuse of information by armed groups, rather than an absence of psychosocial need.

To further strengthen the comparative interpretation, Table 3 presents the thematic analysis of recurrent service adaptations identified across both settings. This analysis shows that the EMT BHSR’s response relied on integrated adaptation across the continuum of care, including primary care, diagnostics, maternal health, public health, referral, specialist care and culturally adapted patient and community engagement. The thematic analysis also highlights that the added value of the EMT model in these contexts lay not only in service availability, but in the ability to reconfigure delivery of care according to changing operational, epidemiological, legal and sociocultural conditions.

The thematic analysis indicated that service adaptation relied on the combined use of the surge capacity domains rather than isolated adjustments within a single domain. Across both displacement contexts, the most consistent patterns included strengthening continuity of care, expanding diagnostic and specialist capacity in remote settings, reducing geographic and sociocultural barriers, and reinforcing referral and public health functions. These findings suggest that adaptability in forced displacement settings depends on integrated service models capable of simultaneously addressing clinical, logistical, and contextual constraints.

Several features underscore the operational strengths of the EMT BHSR model. These include culturally adapted communication and community engagement, particularly with Afro-Colombian and Indigenous populations; the routine use of operational and epidemiological data to inform prioritization and service delivery; and the ability to reorganize care across different crisis settings through mobile deployment, inter-stakeholders’ coordination and collaboration, and flexible team composition. Collectively, these elements strengthened both continuity of care and operational responsiveness across the two displacement contexts.

## 4. Discussion

The EMT BHSR played a critical role in delivering life-saving and primary health services to displaced, confined, and isolated communities in Colombia’s Pacific region and to Venezuelan migrants in transit through Colombia. In both contexts, the teams supported continuity of essential health services where the local system was partially or completely disrupted. Operating amid the presence of armed-groups, pandemic-related isolation, widespread service disruption, and severe food insecurity, the teams provided a broad package of care, including communicable disease management, surveillance for notifiable diseases, maternal and child health, MHPSS, nutrition support, chronic disease management, WASH promotion, and protection services. Innovation and digital health solutions were central to service continuity.

Differences in service demand across the two displacement contexts reflect distinct epidemiological profiles, population mobility patterns, and access barriers. Among Venezuelan migrants in transit, short-term humanitarian needs were shaped by mobility, temporary settlement conditions, and interruptions in access to basic services during migration journeys. This partly explains the higher demand for dental care, chronic disease stabilization, sexual and reproductive health services, and public health education. By contrast, internally displaced communities in the Pacific region experienced prolonged confinement, geographic isolation, and severe disruption of local health services, which contributed to higher demand for clinical consultations, laboratory testing, maternal health services, and community-based health interventions.

Using the 4 domains of the Surge Capacity Framework, this study illustrates how the medical teams constantly adapted health services, staff competencies, medical supplies, and support systems to highly variable and geographically dispersed needs. Key adaptations included flexible clinic schedules to align with migrant mobility, expanded stock for sexual and reproductive health and chronic diseases management; daily operational debriefings to recalibrate services; intercultural staff training with local educators; and the use of local translators in Indigenous communities. These operational adjustments enabled the teams to respond to rapidly changing epidemiological and social conditions while maintaining access to care in geographically dispersed settings.

Trust-building and culturally adapted engagement strategies proved central to service delivery. Collaboration with community leaders, the use of local translators and mediators, and culturally informed communication helped reduce mistrust and improve service uptake, particularly in Indigenous and Afro-Colombian communities. At the same time, risk communication and community engagement played an important role in safeguarding health personnel and facilitating negotiations to ensure access in complex security environments.

The integration of portable diagnostic equipment, telemedicine, and digital health tools during the COVID-19 pandemic further supported service continuity. These technologies enabled monitoring and follow-up of chronic conditions and facilitated clinical decision-making in settings where access to referral facilities was limited. Strengthened referral coordination with local authorities also contributed to maintaining continuity of care despite constrained health infrastructure. Dentistry, although not required for EMT Type 1 classification, emerged as an essential component of care due to the high burden of untreated oral disease and its implications for nutrition, infection risk, and overall wellbeing.

The experience of the EMT BHSR illustrates how health service needs and access barriers evolve along the displacement trajectory under distinct legal and policy environments. Service adaptation, therefore, required flexible delivery models capable of responding to changing epidemiological patterns, mobility dynamics, and contextual constraints. The floating hospital platform enabled the simultaneous provision of onboard clinical services and mobile outreach, extending care to remote communities and migrants in transit or displaced population.

These operational adaptations were reinforced by efforts to strengthen staff empathy, cultural competence, and human-centered care, elements that reduced access barriers and aligned services more closely with the lived realities of displaced populations. Navigating heterogeneous legal frameworks, including IHL, IHRL, Refugee Law, and domestic regulations, further shaped access conditions and underscored the need for legally informed operational strategies to safeguard continuity of care.

At the same time, the intervention revealed operational constraints typical of complex humanitarian settings. Security conditions, mobility restrictions, limited referral options, and variations in local governance arrangements required continuous negotiation and operational adjustments. These constraints underscore the balance between standardized EMT operational models and the need for context-specific flexibility when responding to protracted displacement crises.

By analyzing the EMT BHSR dual response through the WHO EMT Surge Capacity Framework, this study provides a structured analysis of how services, staffing, infrastructure, and systems can be adapted to diverse displacement contexts. Linking this framework with operational experience allows the identification of concrete practices that support service continuity and improved access to care for displaced populations.

Finally, this study, grounded in an operational experience from the Global South, further demonstrates how context-specific innovations developed in resource-constrained and high-mobility settings provide transferable lessons relevant for the improvement of WHO EMT’s standards of care, ensuring that international frameworks remain applicable and flexible to respond to contemporary displacement crises.

## 5. Conclusions

This study demonstrates that Emergency Medical Teams including mobile health infrastructures can play a critical role in responding to forced displacement by combining flexible operational models, community engagement, and multisectoral collaboration to address complex and overlapping humanitarian needs. The findings highlight how service delivery models and standards of care must adapt to displacement realities, including integrating MHPSS, dentistry, RCCE/cultural mediation, specialized ANC for high-risk pregnancies, as well as developing flexible structures and teams capable of mobilizing along migratory routes and across borders, considering the inclusion of mass gathering and mass casualty standards of care, events that can be present during forced displacement crises.

Grounded in operational experience from the Global South, this study shows how context-specific innovations in resource-constrained, high-mobility settings can inform the ongoing evolution of WHO EMT standards of care. By explicitly applying the WHO EMT Surge Capacity Framework in practice, the EMT BHSR response demonstrates how services, staffing, infrastructure, and systems can be adapted in real time to meet the needs of displaced populations. Adaptable service delivery models, supported by tools such as portable diagnostics and telemedicine, can strengthen continuity of care in hard-to-reach environments, while culturally informed approaches help reduce access barriers among displaced populations. Navigating complex legal frameworks, such as IHL, IHRL, Refugee Law and domestic legislations, requires operational flexibility to uphold the right to health and continuity of care. These findings contribute to current reflections within the WHO EMT Initiative on how standards can remain relevant, equitable and adaptable to contemporary displacement crises.

## 6. Limitations

This study presents several limitations. First, as a case study, the analysis focuses specifically on the operational experience of EMT BHSR. The team operated in Colombia’s south-western Andean–Pacific corridor, which does not geographically overlap with the main Venezuelan entry routes in the north-east. Consequently, the Venezuelan response described here reflects only the activities implemented by EMT BHSR within its assigned sector of a broader inter-agency response coordinated by the Colombian health authorities. Because the migrant population in this corridor was highly mobile and often seen only once during short transit stops, some health conditions may be underrepresented in EMT records, particularly as other organizations were simultaneously providing services along the migration route. Second, the comparison between Colombian IDPs and Venezuelan migrants reflects distinct displacement dynamics: IDPs were largely territorially confined and exposed to recurrent insecurity and restricted access to services, whereas Venezuelan migrants were predominantly mobile and able to access humanitarian corridors. These differences may have influenced service utilization and care-seeking patterns. Third, the analysis relied primarily on operational and epidemiological data generated during service delivery. Although triangulated with policy reports and institutional documents, the use of organizational data may introduce reporting biases. Finally, the study represents a cross-sectional analysis of service adaptation during a defined operational period and does not assess the longer-term impact of the intervention.

## Figures and Tables

**Table 1:** Comparative analysis using the WHO EMT Surge Capacity Framework.

Surge Capacity 4 “S”	WHO Standards of Care for EMT Type 1 Fix and Mobile	Venezuelan Forced Displacement Crisis	Colombian Internal Displacement
Services	- Field triage - Trauma assessment, stabilization, and referral - OPD: NCDs, IDs - SRH: antenatal care and basic obstetric care - Pediatric: including screening for Malnutrition - Vaccination - MHPSS - Lab: outpatient testing, rapid tests, no medical imaging - RCCE * * - IPC measures according to capabilities * * - Referral system in place	- OPD: NCDs, IDs with on-site medication dispensing - Pediatric consultation - SRH: Gyn/Obst consultations, family planning, SGBV, Antenatal care including to undocumented migrants - MHPSS - Laboratory * * - RCCE: education campaigns (COVID-19 STIs, Dengue), engagement with community leaders to ensure access and safety for the medical team. * * - Vaccination including for COVID-19 * * - Dental services: were reduce to urgent cases due to the COVID pandemic and referred when necessary. * * - Trauma cases were prioritized. * * - Referral system in place	- OPD: NCDs, IDs, with on-site medication dispensing - Pediatrics - SRH: gyn/Obst consultations, antenatal care, SGBV and family planning previous negotiation and acceptance by community leaders (indigenous communities) * * - MHPSS * * - Lab: Level 1 and Level 2 [30] * * - RCCE; translation to local languages through cultural mediators, health promotion and WASH including educational activities * * - Trauma care was prioritized due to the conflict and confinement. * * - Demand for communicable disease due to overcrowding and poor hygiene: heightened spread of respiratory infections, acute diarrheal disease, tuberculosis, and skin conditions.
Staff	Personnel for PHC: Communicable and non-communicable diseases, basic SRH, basic emergency obstetric and newborn (B-Emoc). Treatment of trauma and non-trauma emergencies, stabilization and referral.	- 3 General Practitioners (GPs) - 2 Psychologists - 1 Social Worker - 1 Registered Nurse - 1 Pharmacy Assistant - 2 Nursing Assistants (support roles) - 1 Obstetrician–Gynecologist who participated specifically to support referrals of high-risk pregnant women without regularized status and lacking access to health services) - 2 Vaccinators from the Cali Health Secretariat who accompanied deployments for COVID-19 vaccination - 1 Head of Mission * * *Training*: - Team received prior training on the response plan, biosafety and security protocols, and preparation for working in multicultural environments with vulnerable populations. * * - Staff wellbeing was supported through rotation schedules. * * - Daily debriefings were conducted to review cases and prepare next day’s operations. * * - Teams were trained to manage verbal aggressions. * * - Staff learned about community costumes, improved to communicate sensitive information.	- 5 General Physicians (GPs) - 1 Pediatrician - SRH: 1 Gyn/Obstetric (MD) - 2 Registered Nurses - Lab: 1 Bacteriologist - 1 Lab technician - Dentistry: 1 Dentist, 1 oral hygienist - 2 Psychologists - 1 Social Worker - 1 Ethno-educator (+translation native language Wounaan) - 1 Registration Technician - 3 Nursing Assistants for screening, triage, and general support - 9 Crew Members (Captain, Boatswain, Machinist, 4 Sailors, 2 Cooks) - 1 Head of Mission (background Epidemiologist) * * *Training:* - Intercultural training was provided by the ethno-educator to ensure staff were sensitized to cultural norms and aware of linguistic and social barriers. * * - A Waunaan-language dictionary with key clinical questions and phrases was developed for interactions with Indigenous communities. * * - For Afro-descendant communities, the team received guidance on local expressions, customs, and cultural practices. * * - Daily debriefings were conducted to review caseload patterns and prepare for the next day’s operations. * * - Staff wellbeing was supported through rotation and debriefings.
Structure/*Space* supplies	Light and portable Able to work in remote areas Operating from suitable existing structures or own tents or equipped vehicles as mobile medical clinics. Self-sufficient in terms of supplies-supply chain, sterilization.	- Inflatable tents fully equipped to ensure medical care. * * - Mobile medical teams: for roadside assistance (migrants on the move), this mobile unit consisted of a truck/van carrying stretchers, tables, chairs, medical supplies and pharmaceuticals. * * - A single tent was used for the mobile operation and moved between different locations. * * - Deployment sites: Cali, Jamundi (Valle del Cauca Department) and Mondomo (Cauca Department). * * - *Supplies*: were adapted to meet the needs for *migrants on the move:* increased supplies of antifungal medications, sunscreen, and analgesics, collyriums/and antibiotics for ocular infections. * * - Chronic patients received a 3-month supply of medications to ensure stability until reaching their destination. * * - Pregnant women showed a high incidence of sexually transmitted infections, including gestational syphilis, which required increasing stocks of relevant treatments. - Long-term contraceptive methods, particularly subdermal implants.	- Full adaptation of the infrastructure (Space) of the Hospital Boat. - Consultations were done onboard. * * - *Supplies*: Certain medicines were prioritized due to the pandemic, especially respiratory infection treatments and analgesics. * * - Increase in PPE stock due to the COVID pandemic, each patient was supplied with a mask, and staff had to use disposable gowns and disinfection materials continuously. * * - Medication rotation patterns changed due to an increase in patients with uncontrolled chronic diseases lacking access to treatment. * * - Medication stocks increased to ensure 3-month supplies for chronic conditions.
System	Coordination, collaboration	- Conducted migrant population mapping with the Local Health Authority to identify priority neighborhoods for deployment. * * - Used community infrastructure: schools, health centers, and communal houses, as operational sites for service delivery. * * - Carried out pre-deployment assessments with community leaders to coordinate activities and gather cultural and customary information. * * - Implemented digital medical records to ensure real-time information access for field teams and responsible institutions. * * - Established advance referral networks with higher-level health facilities and mapped all available services, including ICU beds, hospitalization capacity, and evacuation support agencies. * * - Coordinated with NGOs and partner organizations to optimize resources and prevent duplication of services.	- Community and armed-groups-imposed restrictions in timetable and schedules: adjusted service to meet requests. * * - Telemedicine was implemented during first months of the pandemic, particularly to prevent decompensation of chronic patients. Allowed for teams to anticipate care to communities with restricted access to care. * * - Ensured provisioning not only of medicines but also of food supplies to support community food security. * * - Reconfigured the overall intervention plan to respond to these operational and contextual constraints. * * - Digital patients’ records. * * - On the ground, coordination through local Health Directorate, community engagement technicians previously trained by the medical teams of BHSR, and local Health promoter where available.

**Table 2:** Comparison of health services provided between November 2020 and May 2021.

Health Services	Venezuelan Crisis	Pacific Region/IDPs
General Medical Consultation	1633	15,703
Nutrition Consultation for Children Under 10	1214	3456
Clinical Psychology Consultation	78	595
Public Health Education (COVID, Malaria, Dengue, STIs, GBV)	5229	2398
Psychosocial Support	1617	0
Clinical Laboratory Tests and Rapid Tests	2706	7999
Family Planning Consultation	747	1385
Maternal Health Consultation (Pregnant Women)	191	981
Dental Consultations and Procedures	1053	0
Support for Gender-Based Violence Cases	0	115
Training of Traditional Birth Attendants	0	105
Training of Community Health Agents	0	32
Provision of Health Posts/Medical Equipment	0	17
Teleconsultation/Telemedicine	0	2379
Early Action and Contingency Planning	420	0
Total	14,888	35,165

**Table 3:** Thematic analysis of recurrent service adaptations implemented by EMT BHSR across the continuum of care, mapped against the WHO EMT Surge Capacity domains.

Thematic Area/Operational Challenge	Adaptation	Surge Capacity Domain (s)	Operationalization and Implication Across the Continuum of Care
Continuity of care for chronic diseases	Screening and medication continuity	Systems/Supplies + Services	*Primary Care:* essential medicines, portable diagnostics, onboard consultations, clinical protocols. Improving continuation of care.
Diagnostic adaptability in remote settings and mobile populations	Portable diagnostics (POCUS, rapid tests)	Supplies + Staff + Services	Trained clinicians, portable tests, onboard diagnostics, improve clinical decision making in hard-to-reach areas.
Access and referral in geographic isolated areas	Mobile-outreach teams, hospital referral coordination	Structure + Staff + Systems + Services	*Access and continuity:* mobile hospital (boat), referral documentation, hospital-referral network. Negotiation health authorities. Coordination mechanism. Reduced barriers and improved continuity of care.
Socio-cultural barriers and adaptation of care	Community mediators and adapted communication	Staff + Systems + Services	*Patient/community engagement*: culturally adapted communication. Increased trust and treatment adherence.
Maternal and reproductive health needs	Antenatal screening and reproductive consultations	Staff + Supplies/Structure + Services	Trained staff, screening kits, private consultation areas, ANC protocols. Improved early detection
Public health surveillance and outbreak prevention and response	Syndromic/Case Surveillance and vaccination	Staff + Supplies/Systems + Services	*Public health:* vaccination teams, surveillance systems, vaccines. Improved early detection and prevention
Expansion of specialists and dental care access	Telemedicine and Dentistry services	Staff + Supplies/Structure + Systems + Services	*Specialist and oral health care:* remote specialists, telemedicine equipment, dental staff and supplies. Expanding access to services that are often unavailable in displacement-remote settings.

## Data Availability

The data presented in this study are available on request from the corresponding author. Selected data supporting the findings are publicly accessible on the study at https://barcohospitalhsr.org/proyectos/ (accessed on 14 November 2025).

## Abbreviations

The following abbreviations are used in this manuscript:

BHSR	Barco Hospital San Raffaele
B-EMOC	Basic Emergency Obstetric Care
COVID	Coronavirus Infectious Disease
EMT	Emergency Medical Team(s)
GBV	Gender Based Violence
GHPI	Global Health for Peace Initiative
GP	General Practitioner
HIV	Human Immunodeficiency Virus
IDPs	Internal Displaced Population
IOM	International Organization for Migration
IPC	Infection Prevention and Control
MD	Medical Doctor
NGOs	Non-Governmental Organizations
PAHO	Pan American Health Organization
PHC	Primary Health Care
SRH	Sexual and Reproductive Health
STI	Sexually Transmitted Disease
WHA	World Health Assembly
WHO	World Health Organization

## Appendix A

Standardized terms, such as international migrants, displaced persons, refugees, and asylum seekers, are well established in the literature and by leading organizations in the migration field. Some of these standardized definitions are found in Table A1.

**Table A1:** Definitions of mobile groups.

Terminology	Definition	Organization
Migrants	The term *migrant* has no universally accepted or legal definition. It is often used to describe people who move across borders voluntarily for reasons other than persecution or serious harm but has also been used by organizations as an umbrella term for anyone moving within or across countries—temporarily or permanently—for various reasons, including work, study, or other purposes. For UNDSA, an *international migrant* is anyone who changes their country of usual residence, excluding short-term travel for leisure, business, medical, or religious purposes.	UNHCR Glossary [15]
International Migrants	An *international migrant* is someone who voluntarily moves across borders for reasons such as work, education, or family reunification, without facing persecution or serious harm, and (unlike refugees), who continues to receive protection from their home government while abroad and upon return.	UNHCR Glossary [15]
Internal Migrants	*Internal migration* is the movement of people—nationals or foreigners—within a country to establish a new temporary or permanent residence, either voluntarily or, in the case of internally displaced persons, due to forced circumstances.	Migration Portal [16]
Irregular/Undocumented Migrants	*Irregular migration* refers to movement outside legal or regulated migration channels, though states remain obligated to protect the rights of those migrating irregularly. This category can include refugees, victims of trafficking or unaccompanied minors.	IOM [17]
Trafficked person	A *trafficked person* is someone recruited, transported, or harbored through coercion, deception, abuse of power, or exploitation of vulnerability for the purpose of exploitation, including sexual exploitation, forced labor, slavery-like practices, servitude, or organ removal.	IOM [17]
Internal Displaced Populations (IDPs)	*Internally displaced persons (IDPs)* are people forced to leave their homes due to conflict, violence, human rights violations, or disasters, without crossing an international border.	IOM [17]
Refugee	A *refugee* is someone outside their country of origin who needs international protection due to persecution, conflict, violence, or serious public disorder, meeting criteria in international, regional, or national law, regardless of formal recognition.	UNHCR [15]
Stateless People	A *stateless person* is someone not recognized as a national by any state, either from birth or due to loss of nationality, and may fall under UNHCR’s protection, though not all are refugees.	UNHCR [15]
